# A long-read sequencing and SNP haplotype-based novel preimplantation genetic testing method for female ADPKD patient with *de novo PKD1* mutation

**DOI:** 10.1186/s12864-023-09593-x

**Published:** 2023-09-04

**Authors:** Cuiting Peng, Han Chen, Jun Ren, Fan Zhou, Yutong Li, Yuezhi Keqie, Taoli Ding, Jiangxing Ruan, He Wang, Xinlian Chen, Shanling Liu

**Affiliations:** 1 Center of prenatal diagnosis, Department of Medical Genetics, West China Second University Hospital, Sichuan University, No17, Section 3, South Renmin Road, Chengdu, China; 2 Laboratory of birth defects and related diseases of women and children, Sichuan university, Ministry of Education, Sichuan, China; 3 Yikon Genomics, Suzhou, China

**Keywords:** Long read sequencing, SNP haplotype, PGT-M, ADPKD, De novo mutation

## Abstract

The autosomal dominant form of polycystic kidney disease (ADPKD) is the most common hereditary disease that causes late-onset renal cyst development and end-stage renal disease. Preimplantation genetic testing for monogenic disease (PGT-M) has emerged as an effective strategy to prevent pathogenic mutation transmission rely on SNP linkage analysis between pedigree members. Yet, it remains challenging to establish reliable PGT-M methods for ADPKD cases or other monogenic diseases with *de novo* mutations or without a family history. Here we reported the application of long-read sequencing for direct haplotyping in a female patient with *de novo PKD1* c.11,526 G > C mutation and successfully established the high-risk haplotype. Together with targeted short-read sequencing of SNPs for the couple and embryos, the carrier status for embryos was identified. A healthy baby was born without the *PKD1* pathogenic mutation. Our PGT-M strategy based on long-read sequencing for direct haplotyping combined with targeted SNP haplotype can be widely applied to other monogenic disease carriers with *de novo* mutation.

**Supplementary Information:**

The online version contains supplementary material available at 10.1186/s12864-023-09593-x.

## Introduction

The autosomal dominant form of polycystic kidney disease (ADPKD) is a hereditary kidney disease that results in late-onset renal cyst development, multisystem disorder, and possibly end-stage renal disease (ESRD) after the fifth decade of life [1]. With an estimated incidence of 1:1000 to 1:400 individuals, ADPKD is the most common single-gene inherited kidney disease primarily caused by pathogenic mutations in *PKD1* and *PKD2* genes, encoding polycystin-1 and polycystin-2, respectively [2–4]. Among these, approximately 85% of cases are caused by pathogenic mutations in *PKD1* and about 15% by the *PKD2* gene [5, 6]. As an autosomal dominant disease, ADPKD patients could pass on the pathogenic mutations to their offspring with a 50% chance, thus placing a heavy burden on families and the society suffering from the disease [7].

To prevent the transmission of pathogenic variants, preimplantation genetic testing for monogenic diseases (PGT-M) is increasingly used in clinical practice [8]. Theoretically, PGT-M is available for any monogenic disorder with identified disease-causing locus [9]. In a general PGT-M cycle, multiplex PCR for genetic markers followed by next-generation sequencing (NGS) or SNP array was applied to related family members to identify informative genetic markers for further haplotyping analysis [8, 9]. The high-risk haplotype linked with the familial pathogenic variant should be determined before the clinical cycle, especially in cases for PKD1 PGT-M where direct detection of mutations at the single-cell level is challenging because of homologous pseudogenes and high GC content. However, in some cases, it is hard to determine the high-risk haplotype for *de novo* pathogenic variant or no probands are available. For ADPKD, an estimated 10–15% of cases are *de novo* mutations or gonad mosaicism in parents [1]. Therefore, establishing a reliable linkage analysis for a prospective parent with a *de novo* mutation in *PKD1* is essential in their PGT-M application.

Different from short-read-based NGS, third-generation sequencing (TGS) represented by Pacific Biosciences (PacBio, USA) and Oxford nanopore sequencing technologies (ONT, UK) could generate long reads to cover longer genomic regions, which is potential for direct haplotype phasing [10–13]. Moreover, TGS technologies can directly read the highly repetitive and high GC regions for more sequences [14].

In this study, we reported a successful application of ONT-based long-read sequencing for a female patient seeking PGT-M with *de novo PKD1* mutation and successfully deduced the high-risk haplotype. The result showed the potential of long-read sequencing for direct reconstruction of individual haplotypes, especially for *de novo* mutation carriers, and facilitate identification of embryo status in PGT-M application.

## Methods

### Genomic DNA extraction and whole genome amplification (WGA)

Peripheral blood samples of this couple and oral mucosa cells from the affected female were collected. Genomic DNA was extracted from both blood samples according to the manufacturer’s instructions (QIAGEN, QIAamp DNA Micro Kit). Whole-genome amplification (WGA) was conducted for oral mucosa cells or biopsied trophectoderm cells (TE) from each embryo using the Multiple Displacement Amplification (MDA) method (QIAGEN REPLI-g Single Cell MDA kit) [15]. Whole-genome products were then purified using DNA Clean-up Kit (CWBIO). For long-read sequencing, high-molecular-weight genomic DNA for the female was extracted and purified using QIAGEN Gentra Puregene Blood Kit. Nanodrop quantitation and agarose electrophoresis were conducted to check DNA quality (A260/A230 and A260/A280 are more than 1.80).

### In vitro fertilization and trophectoderm biopsy

Ovarian stimulation, in vitro fertilization, trophectoderm (TE) biopsy, and later embryo transfer processes after genetic testing were conducted in our reproductive medical center as previously described [16, 17]. Each biopsy (approximately 5–8 cells) extracted from the TE was transferred into MDA lysis buffer separately in PCR tubes for WGA and subsequent testing.

### Sanger sequencing validation for *PKD1* c. 11,526 G > C mutation

PCR amplification and Sanger sequencing were conducted to validate the mutation sites of *PKD1* c. 11,526 G > C for the couple and the WGA products. One pair of specific primers (front primer: GTCTACGCCAAGGACAAGGG and reverse primer: GCTGAGGGGCTGTGGAAG) were designed (Primer 5.0 software) and synthesized (Sangon Biotech, Shanghai). PCR amplifications and subsequent Sanger sequencing were performed as previously described [16] to confirm the mutation, and data was analyzed using ChromasPro software.

### Long read sequencing by Oxford nanopore technology

The DNA extract of maternal peripheral blood sample was applied to sequence using Ligation Sequencing Kit (SQK-LSK109, Oxford Nanopore Technologies) on PromethION. The DNA sequencing library was prepared according to the manufacturer’s instructions. 2 µg of high-molecular-weight gDNA was used for library construction using NEBNext End Repair/dA-tailing module. The gDNA was first end-repaired and then pured by AMPure XP beads. 60 µl of end-prepped DNA was ligated with an adapter using NEBNext Quick T4 DNA Ligase (NEB, E6056), then purified and quantified after ligation. The prepared DNA library, sequencing buffer, and loading beads was then loaded onto the primed flow cell of PromethION and performed sequencing. After 26 h of the sequencing run, the detected active pore for sequencing was less than 40%, thus we conducted a Flush Buffer washing process and at the same time added more DNA sequencing library, then restarted the sequencing run in the same flow cell (Flow cell ID: PAH18304). The total throughput was around 120Gb fastq data.

### Data analysis processing: detection of variation at haplotype level

Here we applied the PEPPER-Margin-DeepVariant pipeline to identify variants with the reference genome [18]. The bioinformatic analysis process used several methods to test highly accurate variation as previously published descriptions. Firstly, single nucleotide polymorphisms (SNP) were found by PEPPER-SNP based on a cyclic neural network from reading comparisons. Then, haplotype alignment files were generated using the Hidden Markov Model (HMM). PEPPER-HP used a single point marker to compare files and cyclic neural network (RNN) to find potential SNPs, Insertions and Deletions (InDels) candidate variants. A Deep variant process was further performed to scan more of the genome. Finally, a Margin process was conducted using the output and alignment file of the deep variant to generate the component stage VCF file.

### Multiplex PCR and short-read sequencing for SNP markers

A total of 187 selected SNPs (98 and 89 of SNPs that are at 5’ and 3’ of the gene respectively)within the 2 Mbp region flanking the *PKD1* gene were selected to conduct NGS for haplotype. Specific primer pairs were designed to amplify segments containing these SNP sites and sequencing library preparation. Using the Illumina Miseq Dx platform, we sequenced the library. Afterward, sequence data classification and bioinformatics analysis were conducted by a localized platform of ChromGO (Yikon Genomics) for SNP haplotyping. The informative SNPs that are homozygous in the spouse and heterozygous in the mutation carrier were selected for linkage analysis.

### Copy number variations (CNVs) analysis for the embryos

Low-depth whole-genome sequencing for the TE samples was conducted to fulfill prospective chromosome analysis. The DNA sequencing library was prepared with no modifications as previously described using a Gene sequencing library kit (Yikon Genomics) [19]. The libraries were sequenced on an Illumina Miseq Dx platform using MiSeq Reagent Kit v3 (150-cycle) (Illumina). Following the run, the FASTQ files were uploaded to the localized platform of ChromGO for CNVs analysis. In each embryo, deletions or duplications greater than 4 M will be reported when reads are aligned. Once the analysis is completed, final results, including QC details and CNVs, can be exported.

### Embryo transfer and prenatal genetic diagnosis

Embryo transfer was conducted in our reproductive medical center after genetic diagnosis. Embryo selection criteria were described in our previous study, mainly considering the quality of the embryos and the genetic diagnosis results [20]. The selected embryo was transferred and successfully resulted in a pregnancy. Prenatal diagnosis was performed in the 18th week of gestation by amniocentesis. To verify the mutated *PKD1* allele and chromosomal normality, we performed Sanger sequencing and chromosomal microarray analysis (CMA) using amniotic fluid cells [21].

## Results

### Patient’s genetic background

**Fig. 1:**
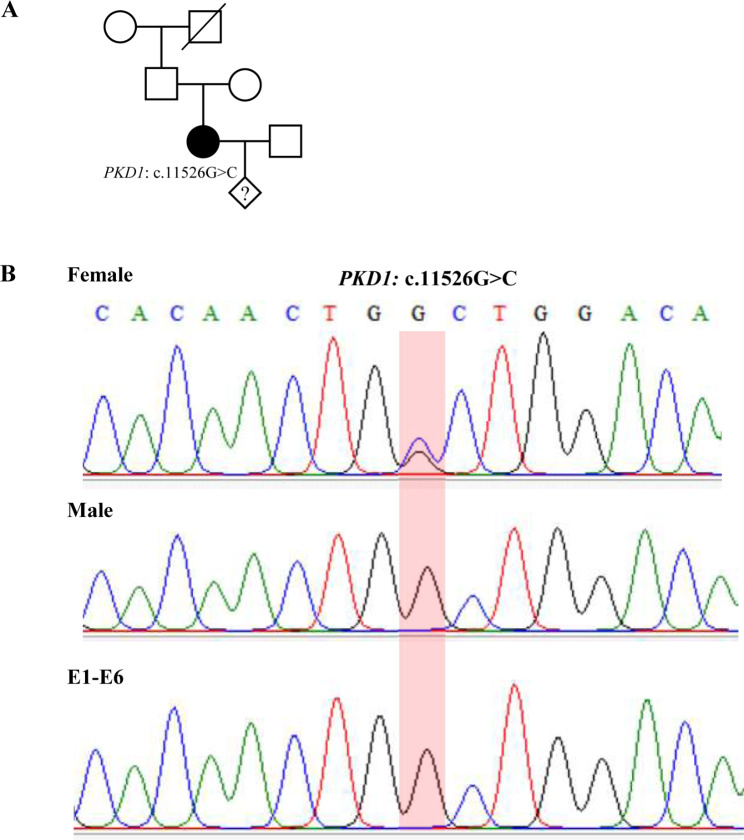
Pedigree of the family (A) and Sanger sequencing for the family and embryos (B). (**A**) In the pedigree, the proband female have the mutation of *PKD1* c.11526G > C but without family history or related family members. (**B**) Sanger sequencing to verify the mutation for the couple and the embryo samples (E1-E6). The single peak in the target sites verified that all the six embryos did not inherit the maternal pathogenic mutation

This study recruited a female patient diagnosed with polycystic kidney disease with adult-onset bilateral cystic kidney. Target sequence capture combined with high-throughput sequencing technology (PANEL) was conducted for the pedigree and identified a *de novo* mutation in the *PKD1* gene (c. 11,526 G > C, p.W3842C) for the female patient (Fig. 1A). According to the guideline of ACMG (American College of Medical Genetics), the mutation was classified as a likely pathogenic variant [22, 23]. The mutation of *PKD1* detected by NGS was then confirmed by Sanger sequencing (Fig. 1B). The couple thus seeks the help of PGT-M to avoid the transmission of the genetic disorder. The study was approved by the Ethics Committee of West China Second University Hospital.

### Direct haplotype phasing for the target region based on long-read sequencing

The extracted high-molecular-weight genomic DNA for the female was used to sequence on PromethION of Oxford Nanopore Technologies. The analysis process was summarized in Figure S1. The mean quality score of base-calling and frequency of nucleotide in reads were evaluated, shown in Figure S1. To obtain sufficient data for haplotype analysis, a sequencing depth of at least 30X was required in the application of PGT-M. Here, 120 Gb fastq data was generated and applied for SNP calling and haplotype phasing. The results of quality control were summarized in Figure S2. A PEPPER-Margin-DeepVariant software was applied to identify variants with long reading length reference genome and conduct direct haplotype phasing for the female. The haplotype results of 4 Mbp flanked *PKD1* c.11,526 (chr16:2141793, GRCh37/hg19) were summarized in an **extended file named chr16 haplotype analysis.** The haplotype with chr16:2141793 G (alternate base) or C (reference base) was defined as maternal haplotype 1 (hap 1) and haplotype 2 (hap 2), respectively. Thus, hap 1 was defined as the maternal high-risk allele based on the ONT sequencing.

### WGA and CNV analysis

**Fig. 2:**
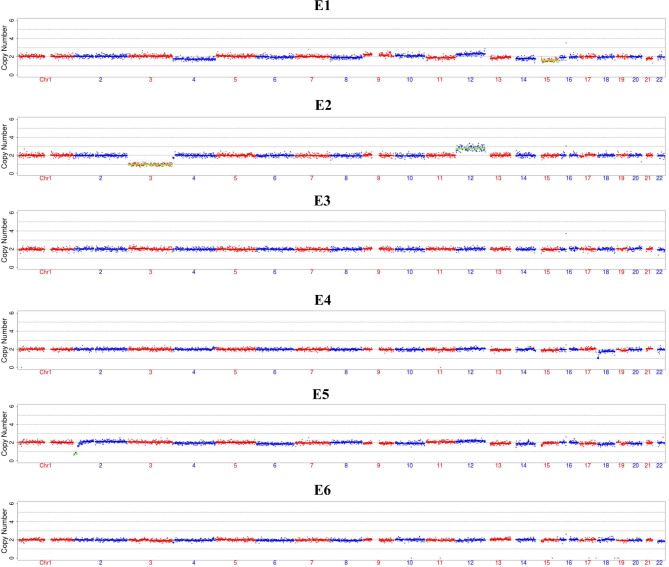
Copy number variations (CNVs) results of the embryos. The sketch maps are generated after compared with the hg19 reference genome through ChromGo. The maps showed that only E3, E6 were euploidy. Other four embryos showed chromosomal abnormalities related to different chromosomes. The detailed CNVs results were presented in Table 1

Trophectoderm biopsy was conducted for three blastocysts on day 5 and three on day 6 post insemination through ICSI in the IVF center. To increase the target gene amplification rate and decrease the allele dropout (ADO) rate, the MDA method was conducted for all six samples [24]. All the samples were amplified successfully and evaluated by the amplification of *GAPDH* as the internal reference gene (data not shown here). Aneuploidy analysis for all the six biopsied blastocysts was performed on an Illumina Miseq Dx platform to detect chromosome CNVs and mini deletion or duplication around 4Mbp. The CNVs results were summarized in Table 1, and sketch maps were displayed in Fig. 2. The results showed that E3 and E6 were normal karyotypes. The other four embryos showed chromosomal number abnormalities or chromosome mosaicism (Table 1).

**Table 1:** Detection results summary of the six biopsied blastocysts

Biopsied blastocysts	Grade of blastocysts	CNV analysis	Sanger Sequencing for c.11,526	SNP raw reads	SNP Mapped reads	SNP mapping_rate(%)
E1	4BC	46,XN,-15(×1,mos,~40%)	Wild type	1,196,354	1,028,405	95.71
E2	4BC	46,XN,-3(×1),+12(×3)	Wild type	1,135,527	978,015	95.98
E3	4BB	46,XN	Wild type	1,025,236	889,159	96.15
E4	5BC	46,XN,-18p(pter→p11.31,~5 Mb,×1)	Wild type	1,322,281	1,141,257	96.06
E5	6BC	46,XN,-2p(pter→p24.3,~16 Mb,×1)	Wild type	1,120,009	976,537	96.36
E6	6BC	46,XN	Wild type	1,262,071	1,063,939	95.t25

CNV: copy number variation detected by low pass whole genome sequencing; SNP: single nucleotide polymorphisms detected by multiplex PCR and targeted NGS.

### Embryo haplotyping analysis based on long-read sequencing and targeted NGS

**Fig. 3:**
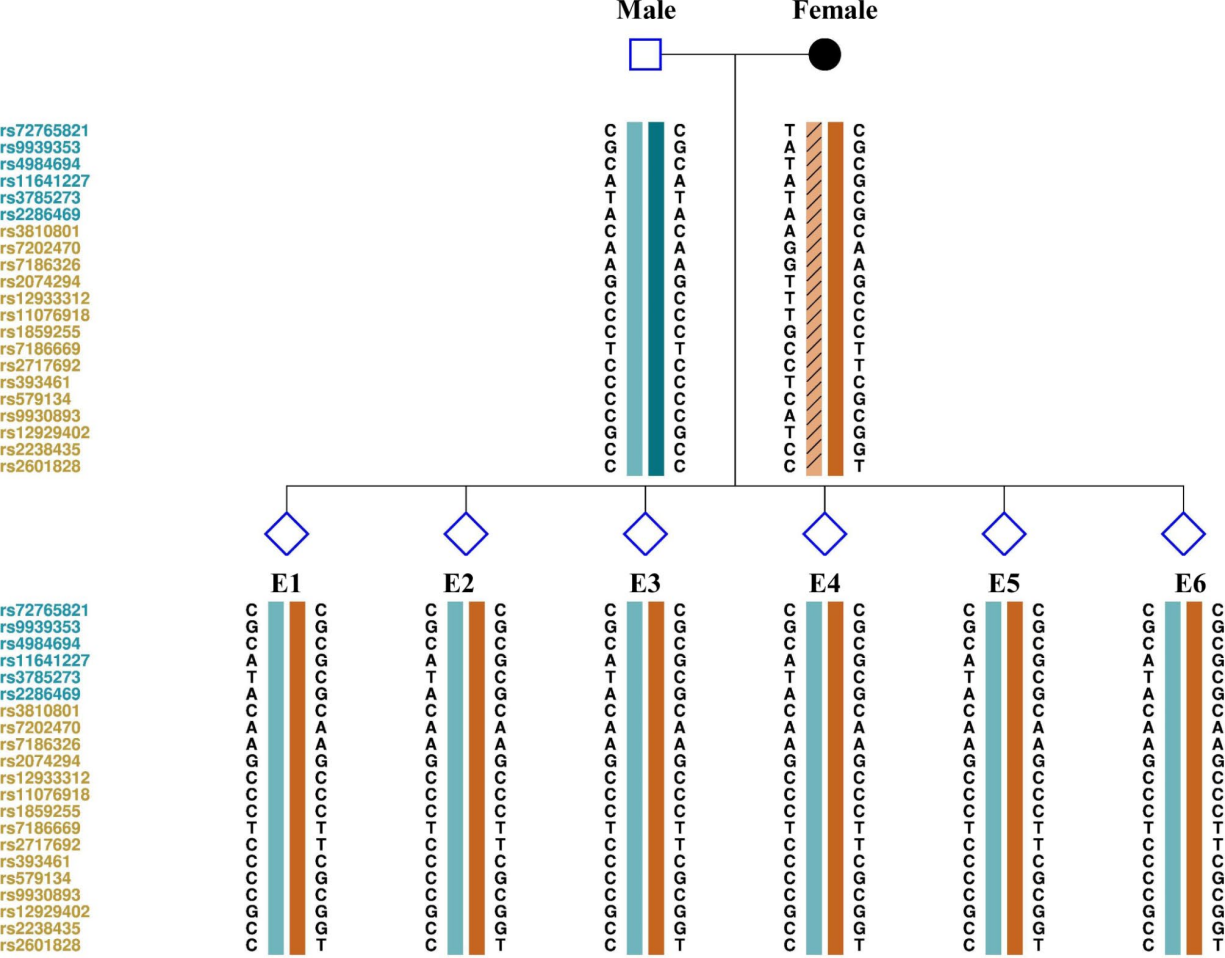
Schematic representative of haplotype linkage analysis for the couple and embryos. Male and female haplotypes were highlighted in different colors. The light yellow frame with slashes refer to the high-risk haplotype of the female deduced by Nanopore sequencing. The reference SNP cluster ID numbers were listed on the left side. The ID numbers highlighted in dark blue and orange refer to the upstream and downstream informative SNPs respectively. All the six embryos (E1-E6) inherited the low-risk haplotype of the female

To avoid the possible misdiagnosis caused by ADO in direct mutation detection using WGA products, the haplotype linkage analysis should be conducted for embryos. Here multiplex PCR and targeted NGS for SNPs around the 2 Mbp region flanking the *PKD1* gene were performed on the couple and embryos. Among 187 SNPs, 25 fully informative SNPs that were homozygous for the male and heterozygous for female were selected for linkage analysis. Additionally, 10 semi- or limited informative SNPs could provide some information for haplotyping (Fig. 3 and Table S1). Together with maternal phasing results as shown in Table 2, all the six embryos had inherited maternal hap 2 .

**Table 2:**
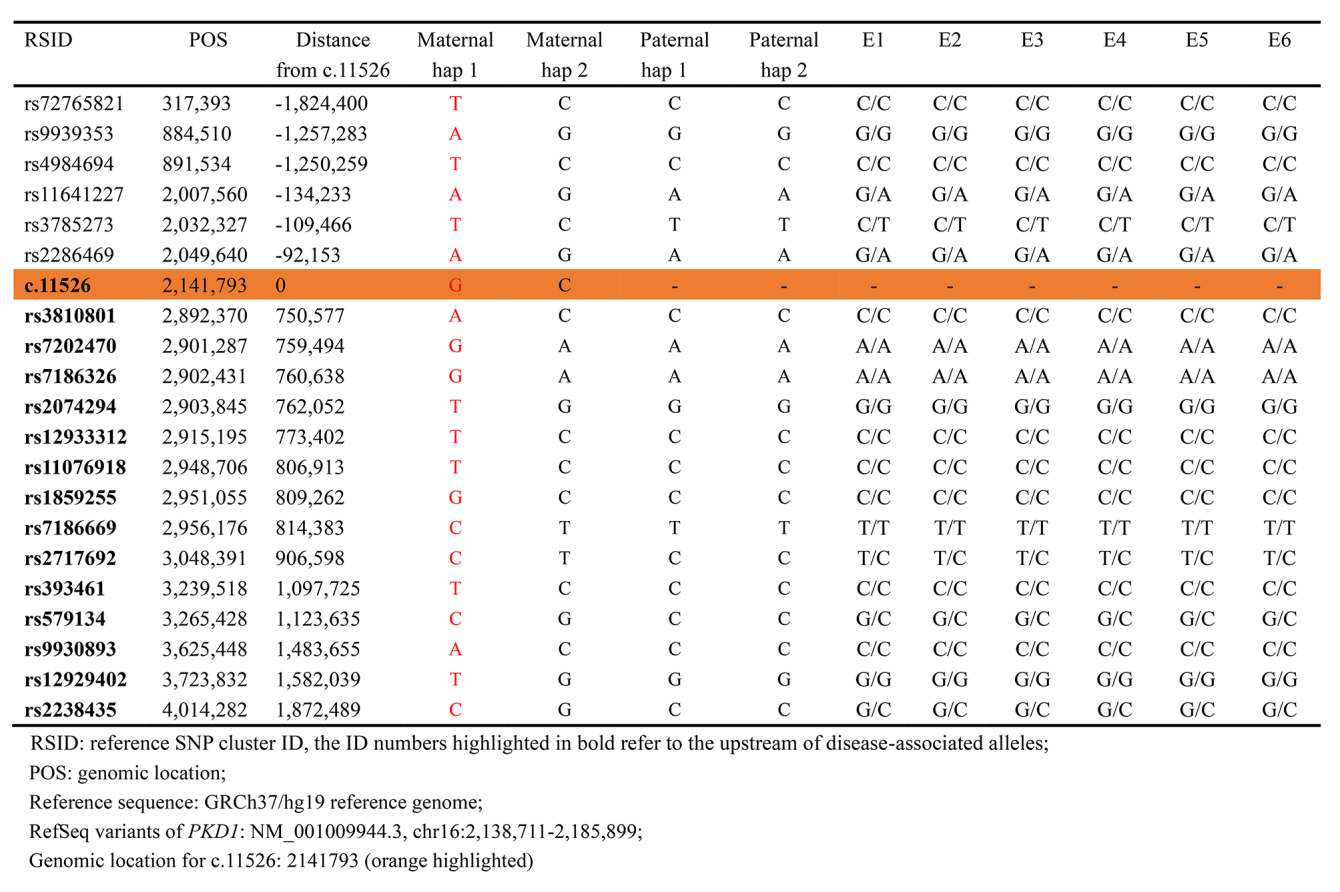
SNP haplotyping linkage analysis based on ONT sequencing and NGS.

Since the site of *PKD1* c.11,526 G > C was not included in the SNP panel for direct next-generation sequencing, it is hard to directly define maternal high-risk allele by NGS. Based on long-read sequencing for the female patient, the pathogenic mutation of *PKD1* c.11,526 G > C was linked to hap 1 as highlighted in red in Table 2. Thus, all the six embryos had not inherited the maternal high-risk allele.

### Sanger sequencing for direct detection of mutation

To further verify the ONT-based haplotype results, direct detection of *PKD1* c.11,526 G > C for each embryo was conducted with targeted PCR according to the same protocol for the blood DNA samples. The PCR product was verified by agarose gel electrophoresis and Sanger sequencing. As shown in Fig. 1B, all embryos (E1-E6) did not inherit the maternal pathogenic mutation of *PKD1* c.11,526 G > C, which is consistent with haplotype analysis results.

### Prenatal genetic diagnosis and pregnancy outcome

Based on the linkage analysis and aneuploidy detection results, only embryos E3 and E6 were identified as normal embryos which can be transplanted. The embryo E3 from day 5 with a better blastocysts score (4BB) was selected to be transplanted and resulted in pregnancy [20]. The prenatal genetic diagnosis was conducted at the 18th week of gestation to validate PGT-M results. Sanger sequencing showed no mutation at *PKD1* c.11,526 and no chromosomal abnormities were detected by chromosomal microarray analysis (CMA). And finally, a healthy baby was born.

## Discussion

ADPKD is one of the most common autosomal dominant polycystic kidney diseases with an estimated incidence of 1:1000 to 1:400 in the population. The couples suffering from ADPKD have a 50% chance of inheriting the disease. Nowadays, the application of PGT-M enables the detection of genetic disorders in embryos and offers an alternative strategy to avoid the transmission of pathogenic mutation. Approximately 85% of ADPKD cases are caused by pathogenic mutations in the *PKD1* gene. Meanwhile, there are six pseudogenes for *PKD1*, which share up to 97.7% sequence identity with the duplicated region of *PKD1* (exon 1–33) [25, 26]. The structural complexity makes molecular analysis of *PKD1* genes challenging, especially in PGT-M application. Thus, pedigree linkage analysis is essential to avoid the misdiagnoses resulting from possible ADO in direct detections. However, for approximately 10–15% of the ADPKD patients with *de novo* mutations in *PKD1* or without a proband, haplotype analysis is challenging. For male ADPKD patients with *de novo PKD1* mutations, an effective haplotype strategy based on single sperm was carried out to analyze the carrying status of embryos [27]. But for female patients, the gametes or PB cells are relatively hard to obtain, which makes haplotype analysis even harder.

**Fig. 4:**
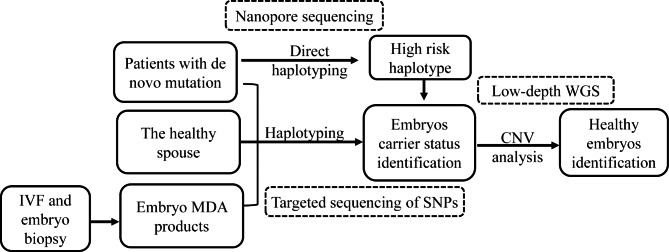
Workflow for long-read sequencing and SNP haplotype-based novel PGT-M method for patient with de novo mutation. Firstly, patient with de novo mutation was conducted Nanopore sequencing for direct haplotype analysis in pre-clinical process. Then, in clinical process of PGT-M, next generation sequencing for the couple and all the embryos was conducted for targeted SNPs. Together with low-depth WGS, the healthy embryos could be identified

In this study, to solve the problem of haplotype analysis for female patients with *de novo* mutation, a long-read sequencing method based on nanopore technology was established for direct haplotyping. Combined with targeted short-read sequencing for the couple and embryos, the carrier status of each embryo could be determined (Fig. 4). Our research established a promising method for *de novo* mutation carrier patients in PGT-M application, especially for female patients. The single sperm haplotype methodology for male patients is usually time and labor consuming because of the high technical demand for single sperm selection, thus also raising the cost for haplotype analysis. For long-read sequencing haplotype analysis, only a blood sample and one nanopore sequencing test are needed for direct phasing in the preclinical process. In addition, targeted nanopore sequencing with designed probes or cas9-guided methodology could obtain more valid data over the targeted region and further reduce detection costs [28, 29].

In the PGT-M clinical cycle for *de novo* mutation, when phasing is unknown at the start of the clinical cycle, at least one affected embryo and one unaffected embryo are needed to establish the correct phase and detect recombination events. Here, an unaffected embryo sample is not recommended to use as phasing reference, since germline mosaicism due to post-zygotic de novo pathogenic variant(s) in the prospective parent cannot be excluded [9]. Yet in our application, all six embryos are absent of the mutation based on Sanger sequencing. Thus direct haplotype is essential for this case. Meanwhile, based on our clinical experience, we found that the MDA whole genome amplification method demonstrated superior efficiency in SNVs detection for repeat regions and regions with high GC content. Thus, the MDA method was chosen for biopsied TE cells to decrease ADO, which guarantees enough mutation site and SNP detection efficiency.

## Conclusion

Third-generation sequencing has exhibited extensive clinical utility applications in many aspects, such as genome assembly, genetic variation detection, and haplotype linkage analysis. Our results based on Oxford Nanopore platform displayed a successful application of third-generation sequencing in PGT-M. Furthermore, this PGT-M strategy could apply to other single-gene disorders with *de novo* mutations or lacking positive family members, facilitating PGT-M implementation.

### Electronic supplementary material

Below is the link to the electronic supplementary material.

Supplementary Material 1

## Data Availability

The datasets generated and/or analyzed during the current study are available from the corresponding author on reasonable request.

## References

[1] Chebib FT, Torres VE (2016). Autosomal dominant polycystic kidney disease: core curriculum 2016. Am J kidney diseases: official J Natl Kidney Foundation.

[2] Torres VE, Harris PC (2009). Autosomal dominant polycystic kidney disease: the last 3 years. Kidney Int.

[3] Heyer CM, Sundsbak JL, Abebe KZ (2016). Predicted mutation strength of nontruncating pkd1 mutations aids genotype-phenotype correlations in autosomal dominant polycystic kidney disease. J Am Soc Nephrol.

[4] Audrézet MP, Cornec-Le Gall E, Chen JM (2012). Autosomal dominant polycystic kidney disease: Comprehensive mutation analysis of pkd1 and pkd2 in 700 unrelated patients. Hum Mutat.

[5] Kurashige M, Hanaoka K, Imamura M et al. A comprehensive search for mutations in thepkd1andpkd2in japanese subjects with autosomal dominant polycystic kidney disease. Clin Genet, 2014. 10.1111/cge.12372 24611717

[6] Mochizuki T, Wu G, Hayashi T (1996). Pkd2, a gene for polycystic kidney disease that encodes an integral membrane protein. Science.

[7] Mir Pardo P, Martínez-Conejero JA, Martín J (2020). Combined preimplantation genetic testing for autosomal dominant polycystic kidney disease: consequences for embryos available for transfer. Genes.

[8] De Rycke M, Berckmoes V (2020). Preimplantation genetic testing for monogenic disorders. Genes.

[9] Moutou C, Dimitriadou E, Dreesen J et al. ,*. Eshre pgt consortium good practice recommendations for the detection of monogenic disorders. 2020*, 10.1093/hropen/hoaa018 PMC7257022 32500103

[10] Jain M, Koren S, Miga KH (2018). Nanopore sequencing and assembly of a human genome with ultra-long reads. Nat Biotechnol.

[11] Logsdon GA, Vollger MR, Eichler EE (2020). Long-read human genome sequencing and its applications. Nat Rev Genet.

[12] Chaisson MJ, Huddleston J, Dennis MY (2015). Resolving the complexity of the human genome using single-molecule sequencing. Nature.

[13] Karst SM, Ziels RM, Kirkegaard RH et al. Enabling high-accuracy long-read amplicon sequences using unique molecular identifiers with nanopore or pacbio sequencing. BioRxiv, 2020, 645903. 10.1038/s41592-020-01041-y 33432244

[14] Li C, Chng KR, Boey EJH et al. Inc-seq: accurate single molecule reads using nanopore sequencing. Gigascience, 2016, 5: s13742-13016-10140-13747. 10.1186/s13742-016-0140-7 PMC4970289 27485345

[15] Dean FB, Hosono S, Fang L et al. Comprehensive human genome amplification using multiple displacement amplification. Proc Natl Acad Sci U S A, 2002, 99. 10.1073/pnas.082089499 PMC122757 11959976

[16] Peng C, Ren J, Li Y (2021). Preimplantation genetic testing for rare inherited disease of mma-cblc: an unaffected live birth. Reprod Sci.

[17] Schoolcraft WB, Treff NR, Stevens JM (2011). Live birth outcome with trophectoderm biopsy, blastocyst vitrification, and single-nucleotide polymorphism microarray–based comprehensive chromosome screening in infertile patients. Fertil Steril.

[18] Shafin K, Pesout T, Chang P-C et al. Haplotype-aware variant calling enables high accuracy in nanopore long-reads using deep neural networks. BioRxiv, 2021. 10.1038/s41592-021-01299-w PMC8571015 34725481

[19] Huang J, Yan L, Lu S et al. Validation of a next-generation sequencing–based protocol for 24-chromosome aneuploidy screening of blastocysts. Fertil Steril, 2016, S0015028216000819. 10.1016/j.fertnstert.2016.01.040 26902859

[20] Dimitriadou E, Melotte C, Debrock S (2017). Principles guiding embryo selection following genome-wide haplotyping of preimplantation embryos. Hum Reprod.

[21] Hu T, Zhu H, Zhang Z (2017). Application of chromosomal microarray analysis for the diagnosis of children with intellectual disability/developmental delay and a normal karytype. Chin J Med Genet.

[22] Cornec-Le Gall E, Audrézet M-P, Rousseau A (2016). The propkd score: a new algorithm to predict renal survival in autosomal dominant polycystic kidney disease. J Am Soc Nephrol.

[23] Richards S, Aziz N, Bale S (2015). Standards and guidelines for the interpretation of sequence variants: a joint consensus recommendation of the american college of medical genetics and genomics and the association for molecular pathology. Genet Med.

[24] He F, Zhou W, Cai R (2018). Systematic assessment of the performance of whole-genome amplification for snp/cnv detection and β-thalassemia genotyping. J Hum Genet.

[25] Berckmoes V, Verdyck P, De Becker P (2019). Factors influencing the clinical outcome of preimplantation genetic testing for polycystic kidney disease. Hum Reprod.

[26] Bogdanova N, Markoff A, Gerke V (2001). Homologues to the first gene for autosomal dominant polycystic kidney disease are pseudogenes. Genomics.

[27] Shi H, Niu W, Liu Y (2021). A novel monogenic preimplantation genetic testing strategy for sporadic polycystic kidney caused by de novo pkd1 mutation. Clin Genet.

[28] Yamaguchi K, Kasajima R, Takane K (2021). Application of targeted nanopore sequencing for the screening and determination of structural variants in patients with lynch syndrome. J Hum Genet.

[29] Gilpatrick T, Lee I, Graham JE (2020). Targeted nanopore sequencing with cas9-guided adapter ligation. Nat Biotechnol.

