# Supplementary material for: A long-read sequencing and SNP haplotype-based novel preimplantation genetic testing method for female ADPKD patient with de novo PKD1 mutation
Source: BMC Genomics. 2023 Sep 4;24:521. doi: 10.1186/s12864-023-09593-x (PMC10478289; doi:10.1186/s12864-023-09593-x)
Supplement: Supplementary file 1 — Supplementary Material 1 [file 12864_2023_9593_MOESM1_ESM.docx]

**Table S1. SNP detected by NGS for the pedigree and embryos.**

| RSID | POS | Distance from c.11526 | Maternal | Paternal | E1 | E2 | E3 | E4 | E5 | E6 |
| --- | --- | --- | --- | --- | --- | --- | --- | --- | --- | --- |
| rs72765821 | 317,393 | -1,824,400 | T/C | C/C | C/C | C/C | C/C | C/C | C/C | C/C |
| rs35533141 | 878,605 | -1,263,188 | C/T | C/T | C/C | _ | _ | C/C | _ | _ |
| rs2974826 | 883,856 | -1,257,937 | A/G | A/G | A/A | A/G | A/G | A/A | A/G | A/G |
| rs9939353 | 884,510 | -1,257,283 | A/G | G/G | G/G | G/G | G/G | G/G | G/G | G/G |
| rs4984694 | 891,534 | -1,250,259 | T/C | C/C | C/C | C/C | C/C | C/C | C/C | C/C |
| rs11641227 | 2,007,560 | -134,233 | A/G | A/A | G/A | G/A | G/A | G/A | G/A | G/A |
| rs3785273 | 2,032,327 | -109,466 | T/C | T/T | C/T | C/T | C/T | C/T | C/T | C/T |
| rs2286469 | 2,049,640 | -92,153 | A/G | A/A | G/A | G/A | G/A | G/A | G/A | G/A |
| **rs8062006** | 2,216,073 | 74,280 | G/C | G/C | G/G | G/C | G/C | G/G | G/C | G/C |
| **rs1541449** | 2,875,483 | 733,690 | C/T | C/T | C/C | C/T | _ | C/C | C/T | C/T |
| **rs4785907** | 2,878,439 | 736,646 | A/G | A/G | A/A | A/G | A/G | A/A | A/G | A/G |
| **rs758189** | 2,881,675 | 739,882 | G/A | G/A | G/G | G/A | G/A | G/G | G/A | G/A |
| **rs12598686** | 2,890,794 | 749,001 | G/A | G/C | C/C | G/C | G/C | C/C | G/C | G/C |
| **rs3810801** | 2,892,370 | 750,577 | A/C | C/C | C/C | C/C | C/C | C/C | C/C | C/C |
| **rs7202470** | 2,901,287 | 759,494 | G/A | A/A | A/A | A/A | A/A | A/A | A/A | A/A |
| **rs7186326** | 2,902,431 | 760,638 | G/A | A/A | A/A | A/A | A/A | A/A | A/A | A/A |
| **rs2074294** | 2,903,845 | 762,052 | T/G | G/G | G/G | G/G | G/G | G/G | G/G | G/G |
| **rs8046218** | 2,908,703 | 766,910 | C/T | C/T | C/C | C/T | C/T | C/C | C/T | C/T |
| **rs12933312** | 2,915,195 | 773,402 | T/C | C/C | C/C | C/C | C/C | C/C | C/C | C/C |
| **rs11076918** | 2,948,706 | 806,913 | T/C | C/C | C/C | C/C | C/C | C/C | C/C | C/C |
| **rs1859255** | 2,951,055 | 809,262 | G/C | C/C | C/C | C/C | C/C | C/C | C/C | C/C |
| **rs7186669** | 2,956,176 | 814,383 | C/T | T/T | T/T | T/T | T/T | T/T | T/T | T/T |
| **rs2717692** | 3,048,391 | 906,598 | C/T | C/C | T/C | T/C | T/C | T/C | T/C | T/C |
| **rs960274** | 3,076,663 | 934,870 | C/T | C/T | T/T | C/T | C/T | T/T | C/T | C/T |
| **rs393461** | 3,239,518 | 1,097,725 | T/C | C/C | C/C | C/C | C/C | C/C | C/C | C/C |
| **rs579134** | 3,265,428 | 1,123,635 | C/G | C/C | G/C | G/C | G/C | G/C | G/C | G/C |
| **rs401877** | 3,312,480 | 1,170,687 | T/C | T/C | T/T | T/C | T/C | T/T | T/C | T/C |
| **rs9930893** | 3,625,448 | 1,483,655 | A/C | C/C | C/C | C/C | C/C | C/C | C/C | C/C |
| **rs12929402** | 3,723,832 | 1,582,039 | T/G | G/G | G/G | G/G | G/G | G/G | G/G | G/G |
| **rs2238435** | 4,014,282 | 1,872,489 | C/G | C/C | G/C | G/C | G/C | G/C | G/C | G/C |
| **rs2601828** | 4,103,871 | 1,962,078 | C/T | C/C | T/C | T/C | T/C | T/C | T/C | T/C |
| **rs1291715** | 4,275,178 | 2,133,385 | T/A | A/A | A/A | A/A | A/A | A/A | A/A | A/A |
| **rs9929311** | 4,510,426 | 2,368,633 | T/C | C/C | T/C | T/C | T/C | T/C | T/C | T/C |
| **rs11076836** | 4,569,331 | 2,427,538 | G/A | A/A | G/A | G/A | G/A | G/A | G/A | G/A |
| **rs2270366** | 4,558,615 | 2,416,822 | G/A | A/A | G/A | G/A | G/A | G/A | G/A | G/A |

RSID: reference SNP cluster ID, the ID numbers in bold refer to the upstream of disease-associated alleles;

POS: genomic location;

Reference sequence: GRCh37/hg19 reference genome;

These semi- or limited informative SNPs are highlighted in orange which could also provide some information for embryo haplotyping when the embryos are homozygous in these SNPs.

**
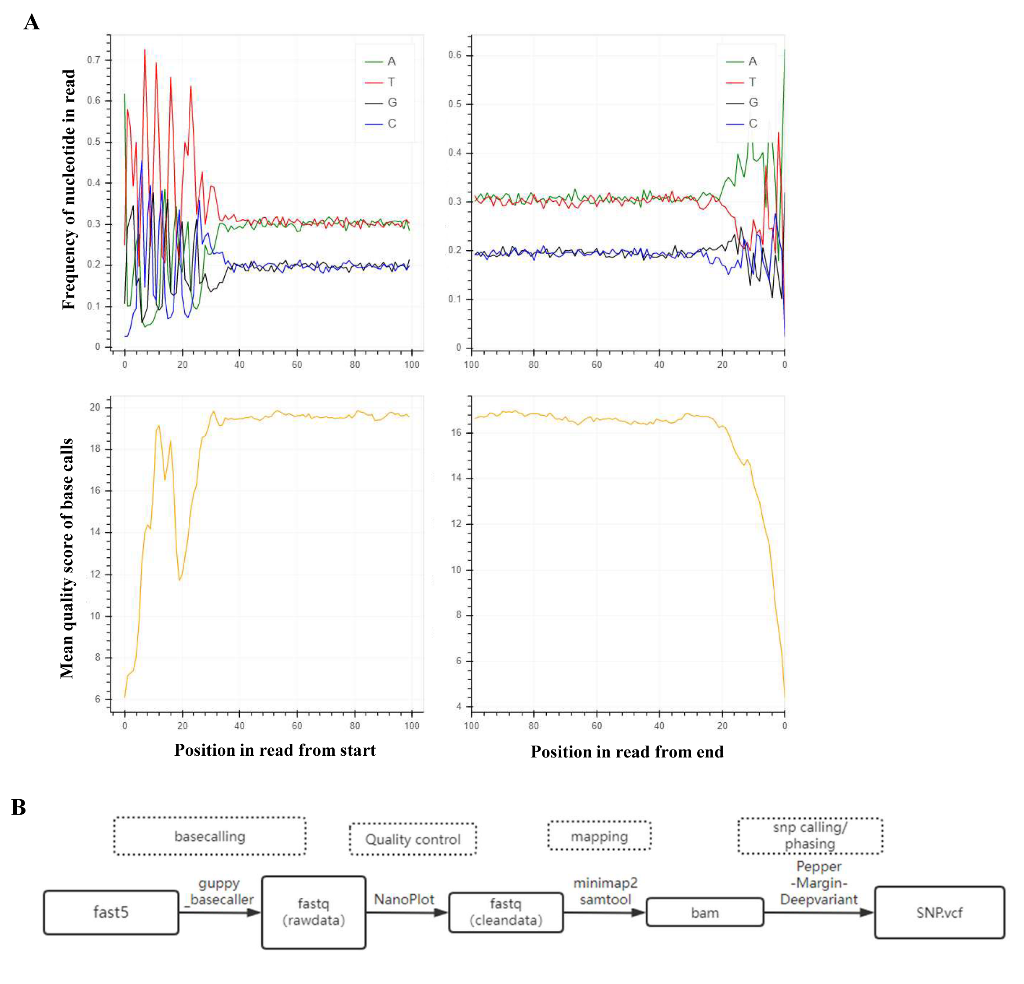
**

**Figure S1. Nanopore sequencing data process.**

Nanopore sequencing data quality control (A) and Schematic diagram of haplotype-aware variant calling using PEPPER-Margin-Deep Variant.


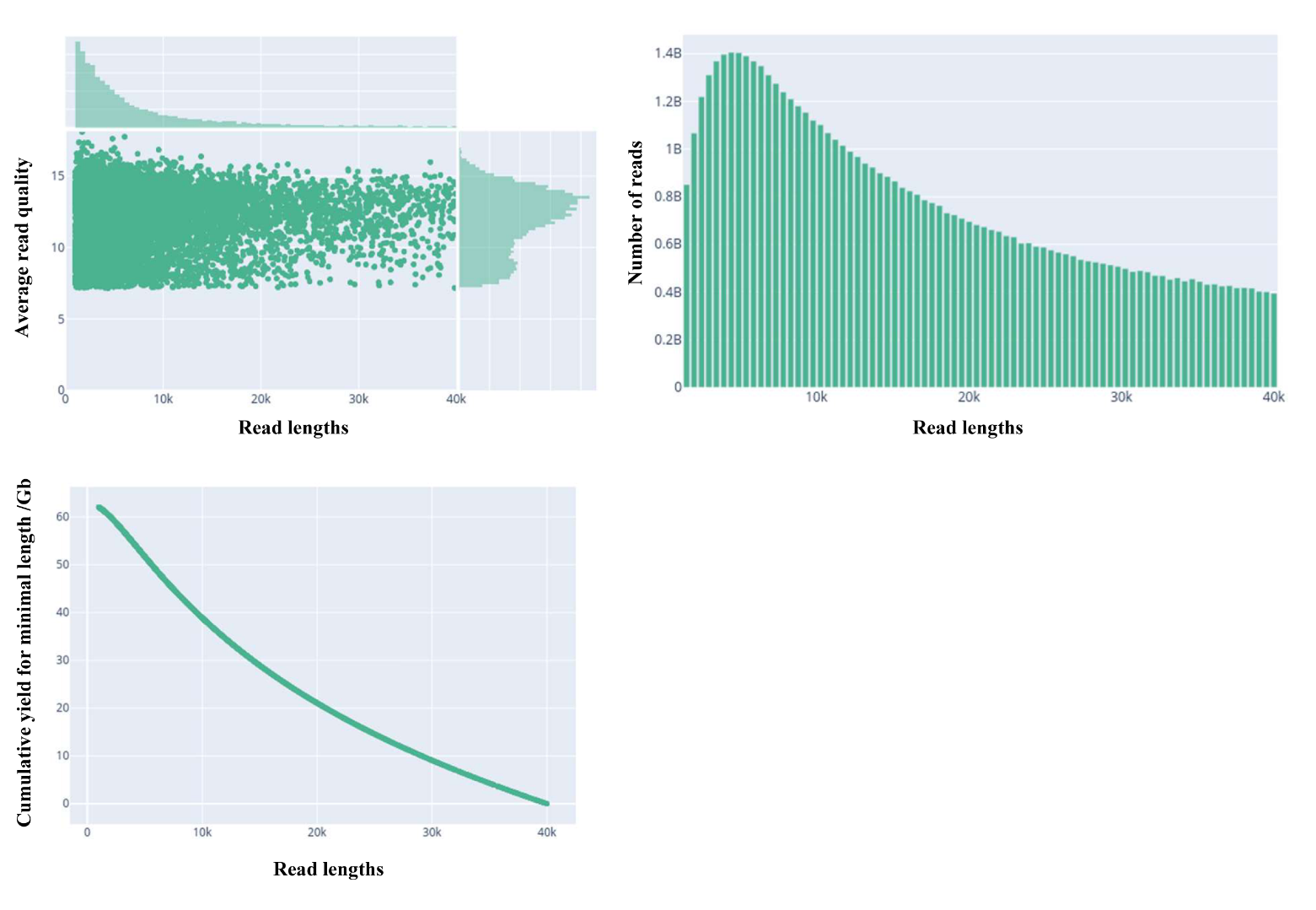


**Figure S2. QC data for ONT sequencing.**

A. Read lengths vs average read quality plot using dots. B. Weighted histogram of read lengths. C. Yield by length.
